# The Impact of Compressed Femtosecond Laser Pulse Durations on Neuronal Tissue Used for Two-Photon Excitation Through an Endoscope

**DOI:** 10.1038/s41598-018-29404-8

**Published:** 2018-07-24

**Authors:** Mira Sibai, Hussein Mehidine, Fanny Poulon, Ali Ibrahim, P. Varlet, M. Juchaux, J. Pallud, B. Devaux, A. Kudlinski, Darine Abi Haidar

**Affiliations:** 1 IMNC Laboratory, UMR 8165-CNRS/IN2P3, Paris-Saclay University, 91405 Orsay, France; 2 Neurosurgery Department, Sainte-Anne Hospital, Paris, France; 3 Neuropathology Department, Sainte-Anne Hospital, Paris, France; 4 IMA BRAIN, INSERMU894, Centre de Psychiatrie et de Neurosciences, Paris, France; 5 Université Lille, CNRS, UMR 8523-PhLAM Laboratory, F-59000 Lille, France; 6 Paris Descartes University, Paris, France; 7 Paris Diderot University, Sorbonne Paris Cité, F-75013 Paris, France

## Abstract

Accurate intraoperative tumour margin assessment is a major challenge in neurooncology, where sparse tumours beyond the bulk tumour are left undetected under conventional resection. Non-linear optical imaging can diagnose tissue at the sub-micron level and provide functional label-free histopathology *in vivo*. For this reason, a non-linear endomicroscope is being developed to characterize brain tissue intraoperatively based on multiple endogenous optical contrasts such as spectrally- and temporally-resolved fluorescence. To produce highly sensitive optical signatures that are specific to a given tissue type, short femtosecond pulsed lasers are required for efficient two-photon excitation. Yet, the potential of causing bio-damage has not been studied on neuronal tissue. Therefore, as a prerequisite to clinically testing the non-linear endomicroscope *in vivo*, the effect of short laser pulse durations (40–340 fs) on *ex vivo* brain tissue was investigated by monitoring the intensity, the spectral, and the lifetime properties of endogenous fluorophores under 800 and 890 nm two-photon excitation using a bi-modal non-linear endoscope. These properties were also validated by imaging samples on a benchtop multiphoton microscope. Our results show that under a constant mean laser power, excitation pulses as short as 40 fs do not negatively alter the biochemical/ biophysical properties of tissue even for prolonged irradiation.

## Introduction

Maximal safe resection is the first line of defence against tumours of the central nervous system^1^. In fact, the extent of resection is a significant prognostic factor, whereby complete or near complete resection can prolong overall patient survival^2^, while also improving their quality of life^3^ compared to patients with sub-optimal resection. A complete resection is typically assessed by comparing the tumour volume that is determined from contrast enhanced T1- and T2- weighted Magnetic Resonance Imaging (MRI) before and after surgery, whereby removing more than 90% of a tumour is considered a near complete resection^4^. The challenge in achieving complete resections is mostly attributed to the necessity of preserving functional tissue, the increased ineffectiveness of pre-MRI based neuronavigation as the surgery progresses due to the increased brain shift and deformations, as well as due to the inability of current intraoperative imaging to identify sparse infiltrating tumours that have migrated beyond the bulk tumour^4^. While the first two challenges are alleviated by functional and anatomical intraoperative imaging, such as tractography and intraoperative MRI where available, state of the art image-guidance cannot delineate diffusive tumours from normal tissue or identify sparse malignant foci that have infiltrated into adjacent tissue. These residuals are the main cause of high relapse rates among patients, where recurrence originates from within 2 cm of the resection cavity^5^. Intraoperative MRI, for example, has a spatial resolution of only 3 mm due to the low magnetic field strength in the majority of the devices and thus has the potential for tumour cells of volumes less than 174 mm^2^ to be left undetected^6^.

Fluorescence-guided resection (FGR) on the other hand, in the form of 5-Aminolevulinic Acid (ALA) induced Protoporphyrin IX (PpIX), is the current standard of care in Europe^7^. It has been a valuable and practical adjunct in achieving safe, complete resection, even beyond the tumour margins defined by MRI^6,7^. However, ALA-PpIX FGR, among other optical techniques^7,8^, presents sub-optimal sensitivities and specificities to efficiently identify low-grade tumours or even diffusive high-grade tumours at the time of resection. That is because available intraoperative optical imaging modalities utilize a broad excitation beam and diffuse remitted light from the tissue; and as a result, they will inherently have a limited spatial resolution in the sub-millimetre to millimetre range^7,8^. Intraoperative confocal microscopy on the other hand, enables visualization of live tissue cytoarchitecture with spatial resolutions from 0.4 to 1.2 µm, yet at the expense of imaging depth and increased photobleaching and tissue damage^9^. Moreover, endogenous structures do not provide sufficient contrasts for confocal fluorescence microscopy and hence exogenous contrast agents are required.

Nevertheless, it is still worthwhile to expand the clinical utility of light-based techniques because of its practicality as well as its unique biophysical properties when applied to the tissue. The unique advantage over other common intraoperative modalities is that the derived intrinsic optical signals can directly relate to the physiological and metabolic state of tissue^10^. Optical measurements should interrogate tissue preferably non-invasively at the sub-micron level and without exogenous contrast, to expoit these advantages optimally. Label-free high-spatial resolution optical imaging can provide the surgeon with a fast microscopic assessment of tumour margins at the surgical cavity as well as from the resected tissue, potentially alleviating the need for time-consuming standard histopathology, while better ensuring no tumour cells are left behind. This is possible using non-linear optical excitation schemes to measure, for example, two-photon emission fluorescence (TPEF) and second harmonic generation (SHG) from endogenous fluorophores.

In non-linear optical imaging, because the distribution of the excitation light must be concentrated in space and time, the non-linear signal is only generated from within a confined tissue volume, and the detected signal is mostly attributed to ballistic photons rather than multiple scattering photons^11^. As a result, two-photon imaging exhibits superior imaging properties compared to conventional linear optical microscopy, where images have spatial resolutions and image contrasts reaching those obtained from confocal microscopy, but with the added benefit of imaging deeper into tissue and inducing significantly less photo-bleaching and tissue toxicity^11^. Although the theoretical advantages of two-photon excitation has been well established for decades, clinical use of two-photon microscopy has only recently gained momentum as pulsed laser systems and dispersion pre-compensation units are becoming more assessable.

As eluded above, shorter excitation pulses increase the probability of two-photon absorption, while maintaining the average power constant. In fact, an inverse relationship between the amplification in TPEF or SHG with pulse length has been proposed^12–14^. However, since shorter pulses increase the laser peak power, it is critical to prevent photobleaching or photo-induced tissue damage within the focal volume. To date, only a limited number of studies have evaluated the photochemical changes that may occur under these excitation conditions. The rate of photo-induced tissue damage *in vitro* was evaluated and compared to typically used longer pulses either by direct monitoring of the fluorescent images with time or by studying cell viability assays post-irradiation^15–20^. Published studies arrived at opposite conclusions; KÖnig *et al*. and Arkhipov *et al*. observed faster photobleaching rates and more cell damage using laser excitation pulses shorter than 100 fs^15,16^, while Xi *et al*., Saytashev *et al*., Pestov *et al*., and Koester *et al*., found that shorter pulses actually reduced cell lethality under the same radiance exposure^17–20^. Since the overall aim is to diagnose and analyze tissue during craniotomy and since ultrafast excitation pulses enhances the efficiency of two-photon fluorescence^12,21^, it is essential to test whether these ultrafast excitation pulses could induce photo-toxicity or tissue damage. Here, we evaluate the potential for neuronal tissue damage of *ex vivo* human brain under different excitation laser pulses by measuring the tissue’s spectral and fluorescence lifetime properties. During these studies, we kept the mean laser power constant typically used in two-photon microscopy for optically thick tissue

Monitoring changes in the characteristic spectral shape and fluorescence lifetimes of common endogenous fluorophores allows one to detect biochemical and metabolic changes that occur in tissue at the sub-micron and cellular levels in real-time, prior to the tissue undergoing macroscopic structural transformations. Specifically, distortion in the fluorescence spectrum and reduction of the emitted fluorescence intensity can indicate photodegradation and photobleaching as it is used to assess the efficacy of photodynamic therapy^22^. In the case of photobleaching, the initial fluorophore undergoes structural changes resulting in having altered spectral absorption and emission properties^22^. Therefore, under the same measurements conditions (NA of the objective, mean laser power, and pulse repetition frequency), any changes in the spectral shape (not intensity) should correspond to changes in the molar absorption coefficient which will only occur in turn if the fluorophore has been molecularly transformed. As the tissue scattering properties at the excitation wavelength may to some degree still affect the generated TPEF signal^23^, changes in the fluorescence lifetimes of these fluorophores are also measured to validate conclusions drawn from the spectral analysis. Since fluorescence lifetime measurements are self-referenced^24^, the derived characteristic lifetime values are insensitive to many of the biophysical factors mentioned above. Changes in lifetime values correspond rather to alterations in the fluorophore’s protein binding properties, its metabolic state, or even changes in the local temperature and pH^24^, all of which occur before cell death. Therefore, the goal is to use the complementary capabilities of spectral and lifetime tissue analysis to ‘sense’ whether compressing the excitation pulse will result in early biochemical changes in the irradiated tissue’s micro-environment as an indicator of photo-induced tissue damage, here as a function of the pulse peak power.

With the present nonlinear endomicroscopic setup built in-house, spectrally- and time-resolved point fluorescence measurements can be performed on *ex vivo tissue* with different short femotosecond excitation pulses at wavelengths ranging from 690–1040 nm. Our custom-built fibre-based endomicroscopic setup is capable of generating excitation pulses from 300 fs down to 25 fs delivered to the distal end of a 5 meter long microstructured double-clad photonic crystal fibre (DC-PCF)^24,25^. The size, the numerical aperture (NA) and the composition of the DC-PCF core and its inner cladding were optimally selected by numerically characterizing -spectrally and temporally- the laser pulse at the distal end of the DC-PCF after propagating through the compression and precompensation unit designed in-house^24,25^. For brain tissue characterization, the fluorophores nicotinamide adenine dinucleotide (NADH), Flavin adenine dinucleotide (FAD), Lipopigments, and Porphyrins are of interest. For this study, two non-linear optical properties of individual endogenous fluorophores are extracted with this setup, TPEF and the fluorescence lifetime, to evaluate whether the desired fluorescence signal enhancement upon irradiating tissue with ultrashort femostsecond laser pulses (<100 fs) would result in tissue toxicity. While a miniaturized MEMS scanning system is currently being integrated into the system for future *in vivo* studies, two-photon and FLIM imaging were performed on the same biopsied samples at the multiphotonic imaging platform for small animals (PIMPA). The motivation behind using the benchtop multi-photon microscope is that FLIM, TPEF and SHG imaging will further validate possible changes detected by endoscopic measurements across a larger field of view, while also spatially assessing metabolic, morphological, and structural changes that may have been undersampled by point measurements.

## Results

### Endomicroscopic measurements at different laser pulse durations

**Figure 1:**
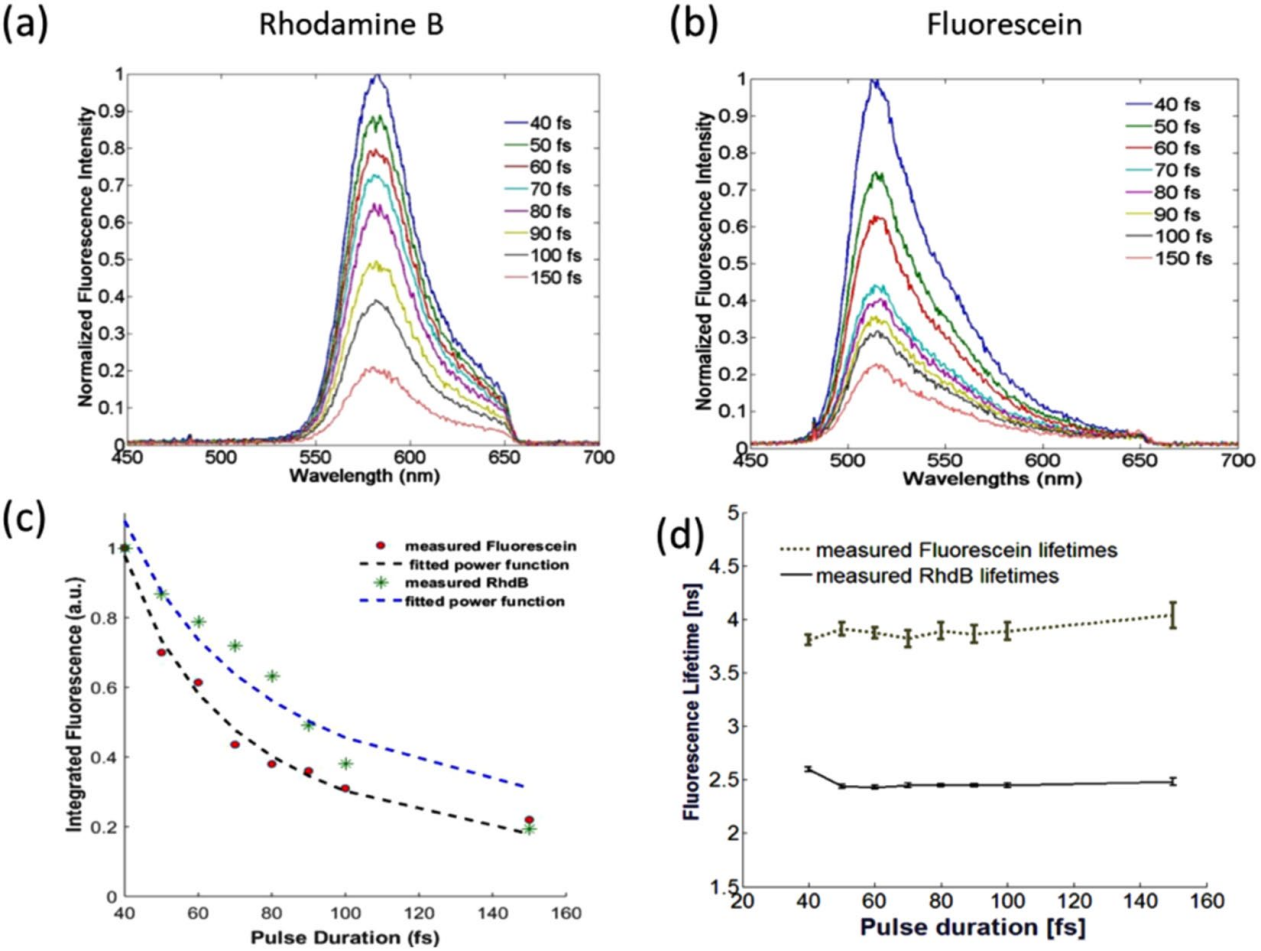
Fluorescence spectra detected by the endomicroscope at 800 nm excitation and at different laser pulse durations for (**a**) Rhodamine B and (**b**) Fluorescein. Each spectrum is normalized to the fluorescence obtained at 40 fs; (**c**) the integrated fluorescence intensity normalized to that measured at 40 fs, while the dashed curve is the corresponding fit to a power function with exponent b. The exponent describing fluorescein’s decay function is 1.28, while that of Rhodamine B is 0.94; (**d**) the mean fluorescence lifetime values (ns) for each dye as a function of pulse duration (fs). Error bars correspond to the standard deviation from five measurements per pulse.

To determine if the fluorescence intensity is a function of the temporal excitation pulse width two standard fluorophores Rhodamine B and fluorescein in aqueous solutions were used. This establishes that the temporal excitation pulse width does not confound lifetime measurements for the different tissue metabolic states and microenvironments. Figure 1(a,b) show the normalized fluorescence intensity spectra of Rhodamine B and fluorescein, respectively, following 800 nm excitation with different laser pulse durations. For both dyes, the spectral shape is not distorted as the pulse duration shortens, but the intensity is positively correlated with pulse duration. The fluorescence intensity is amplified five times when the pulse duration is set to 40 fs as opposed to the conventional pulse width of 150 fs for both dyes. Figure 1(c) plots the mean fluorescence intensity as a function of pulse duration for both dyes. In both cases, the fluorescence intensity decays with increasing pulse duration as a power function with an exponent b = 1.28 and 0.94 for fluorescein and Rhodamine B, respectively. The goodness of fit, R^2^, for each is 0.985 and 0.921. Figure 1(d) on the other hand, shows that the derived fluorescence lifetime values for both dyes remain constant (p > 0.89) at all pulse durations when maintaining the same average power measured at the focal plane.

**Figure 2:**
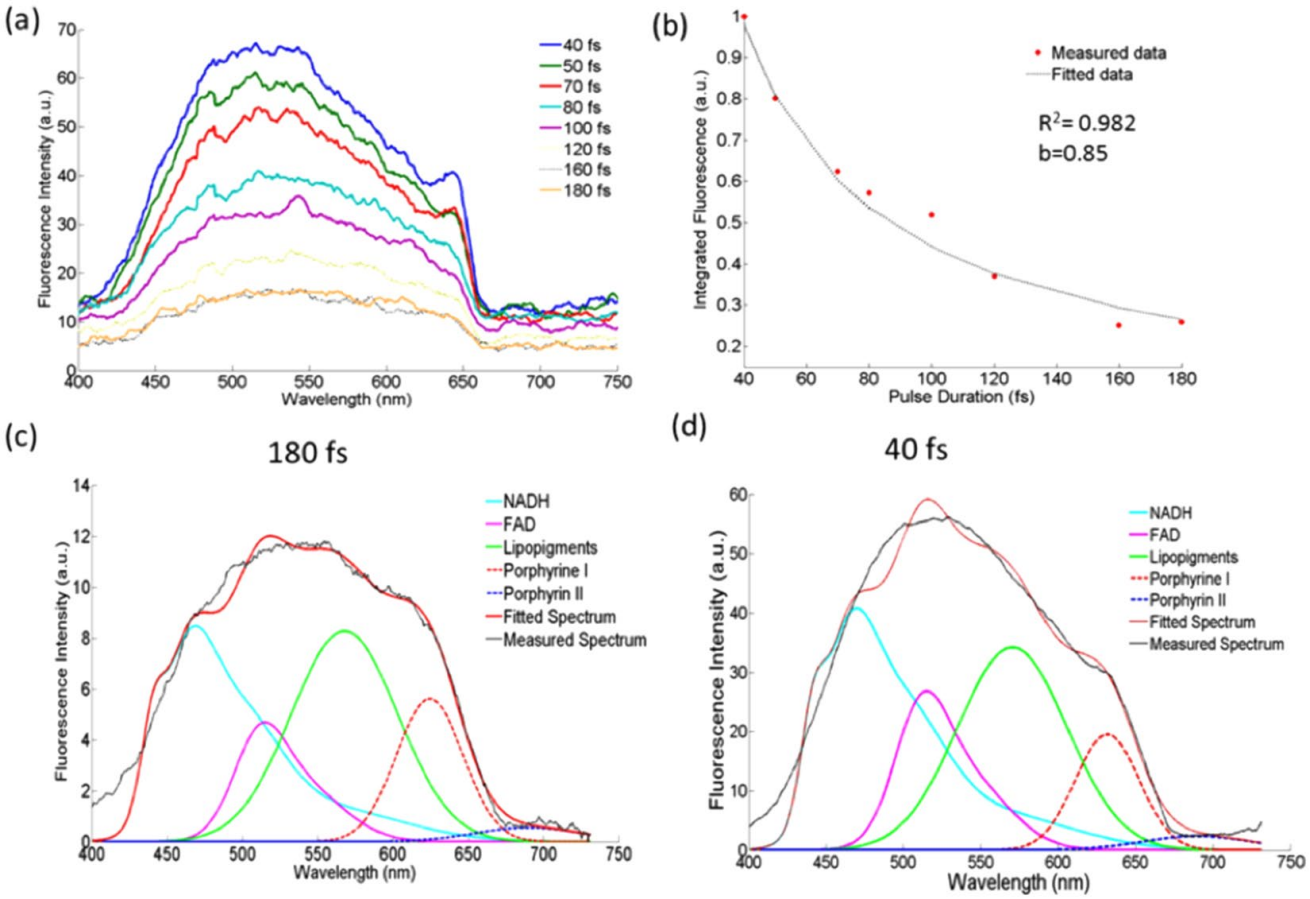
The effect of pulse duration on TPEF obtained from a GBM sample. (**a**) The total autofluorescence spectrum at eight different pulse durations; (**b**) the integrated fluorescence intensity normalized to that measured at 40 fs, while the dashed curve is the corresponding fit to a power function with exponent b; (**c**) spectrally unmixed fluorescence at 180 fs, and (**d**) spectrally unmixed fluorescence at 40 fs.The model fit and measured total fluorescence are also shown for (**c**) and (**d**).

A similar effect of pulse duration on the autofluorescence intensities from *ex vivo* tissue is exemplified in Fig. 2(a–d). The overall fluorescence intensity measured from a glioblastoma multiforme (GBM) sample increases by a factor of 4 when the 180 fs pulse is compressed to 40 fs, while the overall spectral shape is maintained (Fig. 2(a)). The decay in the fluorescence intensity with longer excitation pulses was best fit to a power function with the exponent b = 0.85. Figure 2(c,d) depict the spectrally unmixed fluorescence at 180 fs and 40 fs, respectively, where spectra of individual fluorophore components can be compared. Previous *in vivo* spectral measurements in rats with brain tumours were used as a basis to fit NADH^26^, and the FAD was adjusted to its basis emission spectrum found in the literature^27^, while the remaining three fluorophores were fitted to Gaussian functions. The fluorescence intensity below 420 nm is unaccounted for in our model. However it is attributed mostly to collagen crosslinks^26^, having an absorption peak at 380 nm. Decomposing the fluorescence spectra into the individual fluorophores was necessary to validate whether different pulse durations altered the biochemical state of these five endogenous molecules used in diagnosing brain diseases. The relative contribution of each fluorophore remained constant, indicating that no spectral distortion pertaining to any individual fluorophore investigated had occurred as a result of using shorter excitation pulses. In fact, when comparing the peak intensity of each fluorophore at 180 fs and 90 fs pulse duration, for example, the fluorescence signal amplification was found to be 2.6 ± 0.24. Although Fig. 2 pertains to results from a GBM sample, similar conclusions were found for all available tissue types: healthy cortical tissue, meningioma, and metastatic tissue, where the exponent of the power function was found to be between 0.85 and 1.35.

**Figure 3:**
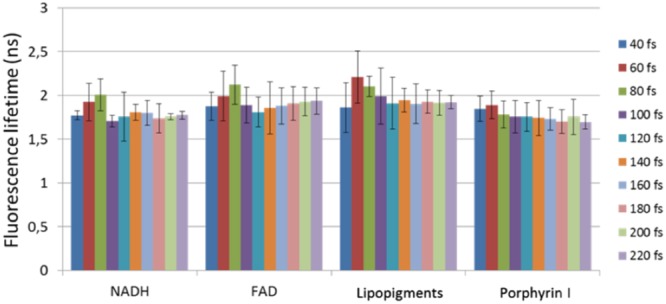
Bar plot showing the mean fluorescence lifetimes for each fluorophore extracted from endoscopic time-resolved fluorescence measurements at different pulse durations. The error bars correspond to the standard deviations pooled from five ROIs per GBM sample.

As a means to gauge possible changes in the metabolic and chemical binding state of the relevant fluorophores in tissue, time-resolved fluorescence was performed following 800 nm excitation. The band-pass filters allowed the extraction of the characteristic fluorescence lifetimes of NADH, FAD, Lipopigments, and Porphyrin I after fitting the decay profiles with a mono/bi-exponential decay fit^26^. As an example, the mean lifetimes recovered for each fluorophore from GBM samples is depicted in the histogram of Fig. 3, where the excitation pulse varied from 40 fs to 220 fs in steps of 20 fs. The error bars correspond to the standard deviations pooled from five different ROI measurements for each of the GBM samples. The deviation in the recovered lifetimes for each fluorophore across all GBM samples did not vary greatly across the region of interests, and more importantly, it did not vary according to the pulse duration applied.

The mean fluorescence lifetimes recovered from all pulse duration measurements for each tissue type is summarized in Table 1. The minimum p-value calculated from each fluorophore per sample is provided in the last column, indicating there was no significant difference in the lifetime values recovered at different excitation pulses (p >> 0.05) for all four tissue types. As mentioned in the methods section, if under the same excitation pulse, five ROIs resulted in statistically different lifetime values for a given fluorophore, a paired t-test was performed for each ROI instead of an ANOVA test pooled from all five ROIs treated with ten pulse durations. This was the case for the lifetime values of FAD and Lipopigments from healthy cortical tissue, as well as the FAD lifetimes from Meningioma and Metastatic samples. Nevertheless, the minimum p-value among all four fluorophores per tissue type was always larger than 0.29, indicating that short and long excitation pulses resulted in recovering similar fluorescence lifetime values at the 95% significance level and a power of 98%.

**Table 1:** Fluorescence lifetime values for each fluorophore averaged across all excitation pulse durations and regions of interests.

Tissue Type	NADH (ns)	FAD (ns)	Lipopigments (ns)	Porphyrin I (ns)	p-value
Healthy Cortex	1.40 ± 0.13	1.53 ± 0.13	1.60 ± 0.15	1.57 ± 0.095	0.81
GBM	1.92 ± 0.13	2.0 ± 0.06	2.0 ± 0.17	1.90 ± 0.079	0.97
Metastatic	1.89 ± 0.14	1.84 ± 0.230	2.02 ± 0.27	3.47 ± 0.012	0.29
Meningioma	1.63 ± 0.095	1.78 ± 0.095	1.92 ± 0.18	3.19 ± 0.67	0.50

The p-values derived from one-way ANOVA analysis or paired t-test analysis of each fluorophore indicate that changing pulse durations did not result in changes in the individual fluorophore lifetimes.

### Two-photon imaging at different laser pulse durations

**Figure 4:**
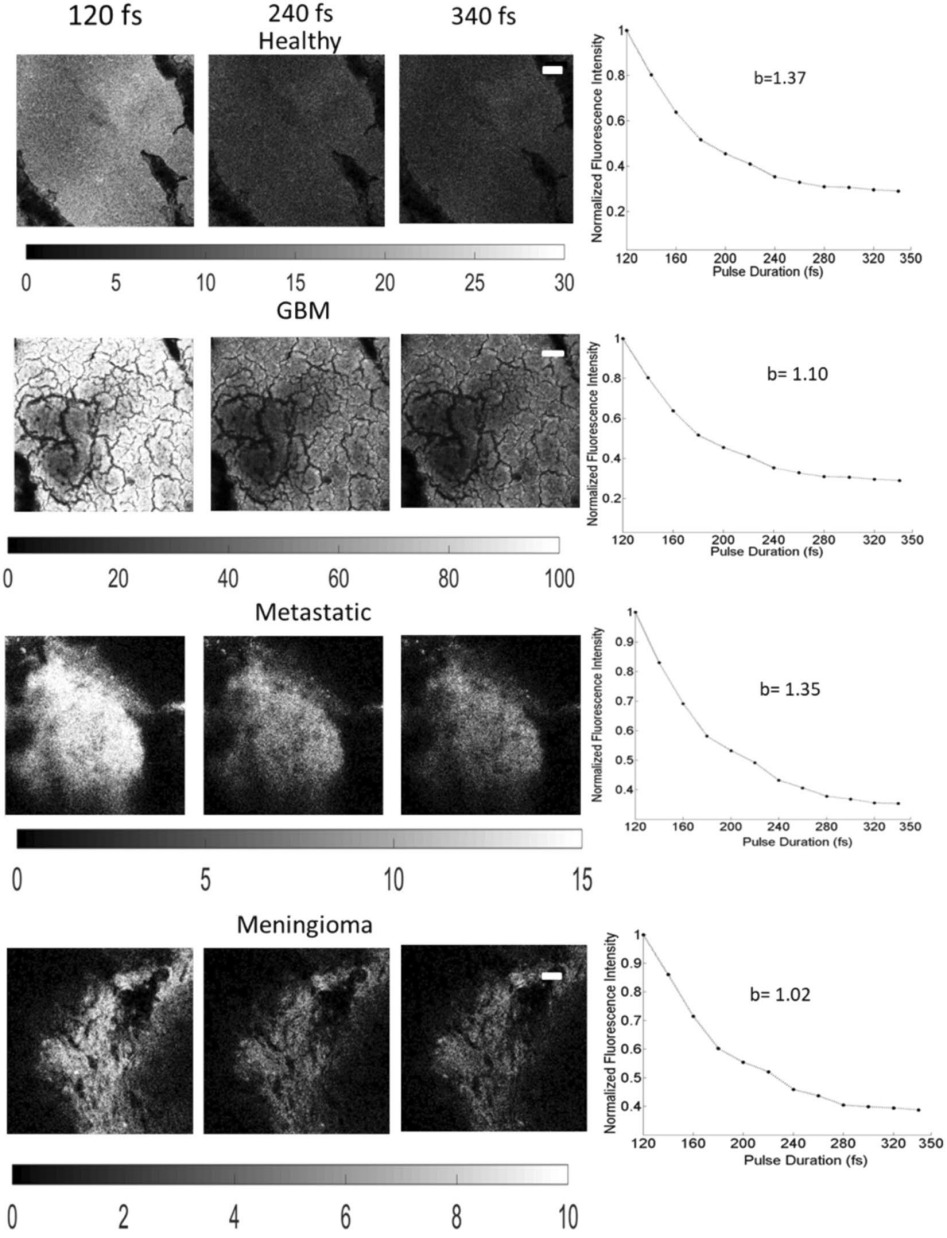
TPEF images from a healthy cortical tissue sample (first row), GBM (second row), metastatic tissue (third row) and meningioma (fourth row) following an excitation of 890 nm and obtained using a 120 (first column), 240 (second column) and 340 fs (third column) pulse durations. The scale bar for all images correspond to 50 µm, and the gray-scale display range for all three images is shown below one of the TPEF images in brackets. The normalized mean intensity from each TPEF image is plotted in the fourth column as a function of the excitation pulse used. The exponent, b, of the fitted power function is also displayed.

Figure 4 illustrates the quality impact of compressing the excitation pulse duration of two-photon images from healthy cortical tissue (first row), GBM (second row), metastatic (third row) and meningioma (fourth row) biopsied samples, respectively. The first three columns correspond to TPEF images taken at 120, 240 and 340 fs following 890 nm excitation 520 ± 30 nm detection. The gray scale colorbar range was kept constant for all three pulse durations per sample as displayed under each image set, so the superior image quality at the shortest pulse is prominent. Two-photon images from GBM and Metastatic samples also showed significant SHG signal (not shown here). The fourth column of each row shows the fluorescence intensity as a function of excitation pulse averaged across each image and normalized to the mean intensity obtained at 120 fs. All curves were best fitted to a power function with an exponent power, b, displayed on the plots. For each sample, the TPEF signal decayed with similar values of b (1.02–1.37). Shortening the excitation pulse by a factor of 2 (from 240 to 120 fs) resulted in an intensity enhancement of at least 2.6 in the TPEF images. TPEF and SHG signals at 800 nm (not shown here) resulted in similar intensity decay profiles to those obtained at 890 nm excitation with the exponent b ranging from 1.1 to 1.4.

**Figure 5:**
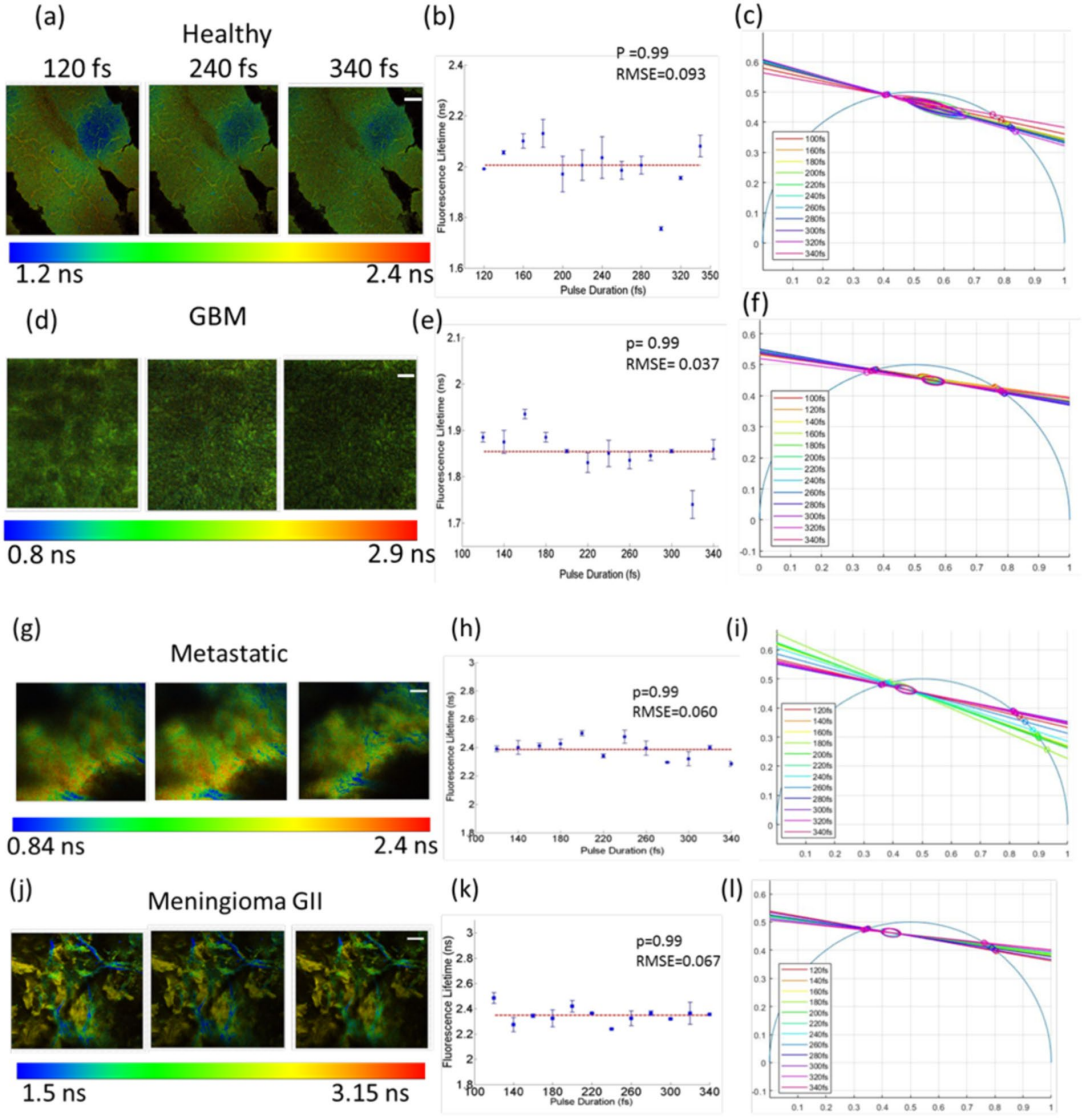
FLIM analysis: (**a**,**d**,**g**,**j**) represent fluorescence lifetime images obtained at three excitation pulses for healthy (first row), GBM (second row), metastatic (third row) and meningioma tissue (fourth row), respectively. The scale bar of 50 µm is displayed in the third column for each tissue type and a horizontal colour bar representing the range of calculated fluorescence lifetimes is shown below the set of images. The corresponding mean fluorescence lifetimes are plotted against pulse duration in (**b**,**e**,**h**,**k**) where error bars correspond to the standard deviations obtained from calculating the values from five ROIs. The root mean square error, RMSE, and p-values > 0.05 indicate no significant difference between the data points based on regression analysis and a paired t-test, respectively. (**c**,**f**,**I**,**l**) represent the phasor distribution of the fluorescence lifetimes and their intersections with the universal circle at pulse durations ranging from 120 fs to 340 fs.

After examining the benefit of shortening the excitation pulse durations towards improved image quality of two-photon imaging with no visual photodegradation, it was necessary to test whether shorter pulses, induced any changes in the biochemical state of the endogenous fluorophores, mainly NADH and FAD based on the available detection filters. This was first assessed by FLIM as shown in Fig. 5. Examples of fluorescence lifetime images at the three excitation pulses are shown (Fig. 5(a,d,g,j)) for each tissue type. Images are displayed with a common fluorescence lifetime colour code since FLIM produced the same extent of lifetime values for all three excitation pulse durations. The distribution of the fluorescence lifetime was extracted by fitting the fluorescence decay profile of a given ROI to a double exponential tail function. The mean lifetimes and corresponding standard deviations were obtained from averaging the values of five ROIs per image and are plotted in Fig. 5(b,e,h,k). The dashed lines in each plot correspond to the mean fluorescence lifetimes recovered at all excitation pulse durations. The data points were fitted to this horizontal line, and the resulting root mean square error, RMSE, is shown on the top right corner of each plot, indicating that no linear increase/decrease in the recovered values was observed as a function of pulse duration. Furthermore, a paired t-test was performed to show that there was no significant difference between the recovered values (p-value = 0.99 and type II error = 0.99). A previously established phasor approach^28^ was also applied to the FLIM data to better visualize the consistency in the recovered lifetime values when the excitation pulse duration varied from 120 fs to 340 fs. The phasor plots in Fig. 5(c,f,i,l) show that the shape of the 95% confidence ellipse is small and corresponds to almost the same two lifetime values on the universal circle for all pulse durations per tissue type.

**Figure 6:**
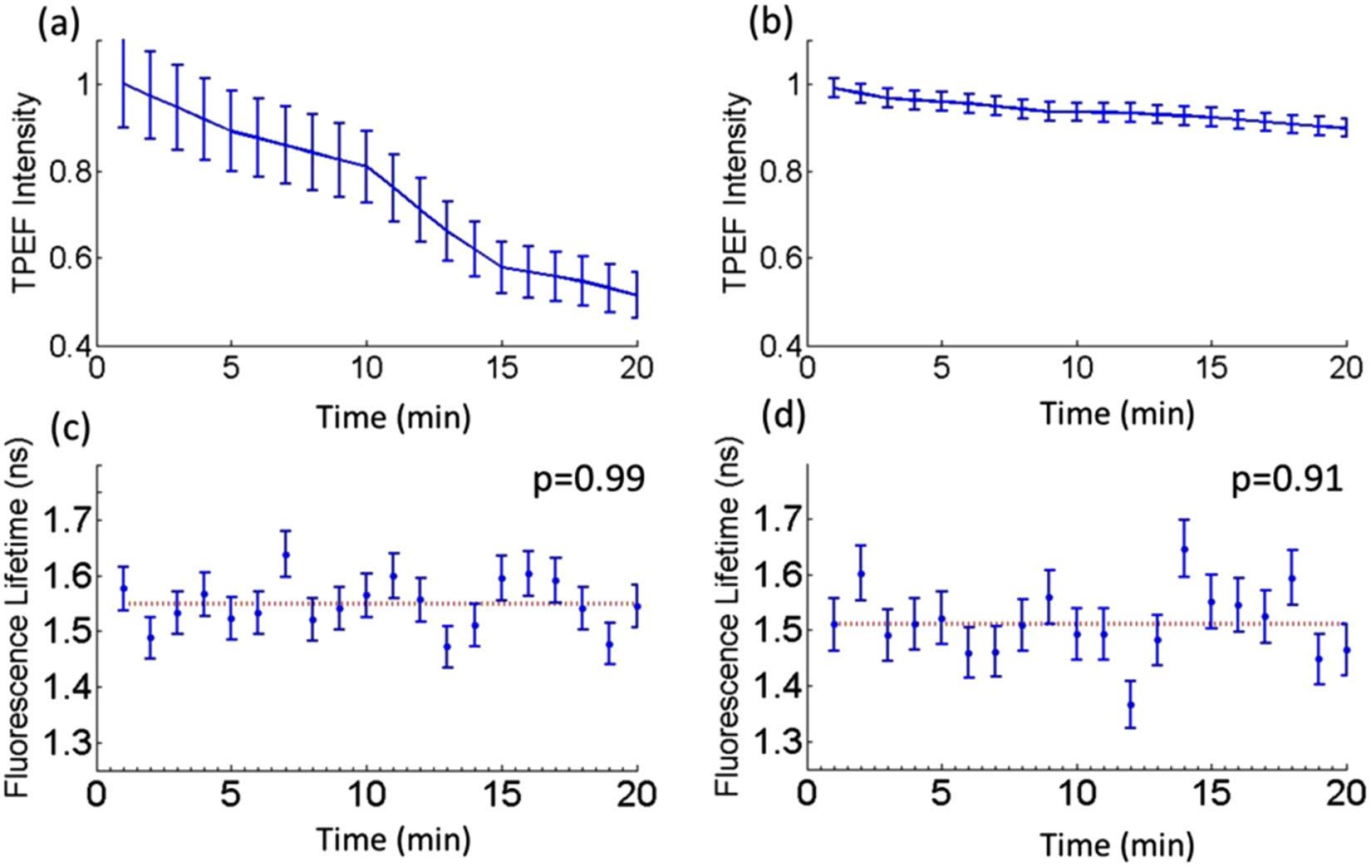
The decay of the TPEF intensity as a function of exposure time at low (**a**) and high (**b**) laser irradiances corresponding to those used on the multi-modal microscope and with the endomicroscope respectively. The error bars correspond to the standard deviation between pixels across a selected ROI. The fluorescence lifetime as a function of time at low (**c**) and high (**d**) laser irradiances. The error bars correspond to the standard deviation in the lifetime values calculated for the selected ROI. The horizontal line corresponds to the overall mean lifetime, and the p-value indicates no significant difference in lifetime values when measured for different exposure times based on a paired t- test.

Since higher laser fluence rates can cause photobleaching, the laser power was tuned to produce the maximum fluence rates used for imaging tissue at PIMPA and the maximum fluence rate that will be used *in vivo* with the endomicroscope. This was found to be 22 × 10^8^ *W/cm*^2^ and 1.0 × 10^9^ *W/cm*^2^ for the multi-modal microscope (at 8 mW with 100 fs excitation pulses) and the endomicroscope prototype (at 38 mW with 100 fs excitation pulses) respectively. The corresponding TPEF decay profiles as a function of exposure time are shown in Fig. 6(a,b). The photobleaching decay rate is observed to be significantly slower at the high irradiance case, where the fluorescence intensity only decreased by 11% after 20 min of imaging (Fig. 6(b)). In both low and high irradiance conditions, a double exponential best fitted the decay curves (R^2^ > 0.98) incorporating a fast and slow photobleaching decay constant. At 2.2 × 10^8^ *W/cm*^2^, the photobleaching half-life period was 18 min and the photobleaching decay constants were 0.78 and 0.024 min^−1^. The photobleaching half-life period on the otherhand of the same sample irradiated at 1.0 × 10^9^ *W/cm*^2^ was estimated to be 200 min and the photobleaching decay constants were 0.8 and 0.004 min^−1^. The mean fluorescence lifetime for each FLIM acquired every minute is also shown in Fig. 6(c,d) after irradiating at 2.2 × 10^8^ *W/cm*^2^ and 1.0 × 10^9^ *W/cm*^2^, respectively. The p-values indicate no significant changes in the lifetime values as a function of exposure time for both cases. Additionally, the mean lifetimes of 1.55 ± 0.39 ns and 1.51 ± 0.47 ns at low and high irradiances are similar, as indicated by the horizontal dashed lines.

## Discussion

Maximal surgical resection of solid tumours can be a curative treatment for most cancer patients without local or distal metastasis. Because of the potential for metastasis, surgery is followed by adjuvant chemotherapy and radiation therapy^2^. However, current intraoperative imaging modalities lack the sensitivity and spatial resolution to detect sparse or diffusive tumours^10^. This is more so the case for brain tumours, as their margins can be indiscernible from normal brain tissue, hence increasing the risk of relapse due to the limited efficacy of chemotherapy in the brain. Accurate tumour margin assessment requires microscopic tissue characterization *in vivo*, in near real-time on the one hand, while also having the ability to analyze the entire resection bed on the other hand. To date, no imaging technology can effectively examine tissue *in vivo* over a large field of view at a cellular level^10^, which is the motivation behind developing a vari-focal non-linear endomicroscope. It was shown that multiple optically accessible tissue properties could be measured and quantitatively correlated to disease biomarkers^24,29^; multi-modal optical measurements will provide quantitative histopathological information *in vivo* and for freshly resected tissues during the course of the surgery, stain-free. To generate sufficient non-linear signals such as two-photon fluorescence and SHG, Fourier-transform limited femtosecond pulsed lasers and a high NA at the objective are required to temporally and spatially focus the beam^11^. The excitation pulse duration is inversely proportional to the molecules’ two-photon absorption cross section and thus shorter excitation pulses are desired for superior image quality, enhanced imaging depth and for faster acquisition times^12,13^. Compressing the pulse duration, however, results in quadratically amplifying the peak laser power at the sample, potentially causing photo-induced damage to the tissue^13–15^. Therefore, as a prerequisite to future *in vivo* studies, it was necessary to study the effect of shortening the excitation pulse duration on tissue microenvironment. The study involved evaluating the spectral and lifetime fluorescence properties of relevant endogenous fluorophores in the brain from fixed tissue samples pathologically classified as healthy cortical tissue, GBM, metastases, or meningioma (grade I or II) as a function of pulse width. Although the endogenous fluorescence properties *in vivo* may differ from that of fixed tissue^29^, measurements were performed on Formalin fixed tissue to separate changes in tissue morphology/structure due to autolysis and putrefaction from that induced by altering the laser pulse width for two-photon excitation. Formalin-based tissue fixation has been shown to minimally alter tissue optical properties as well as the characteristic autofluorescence spectra compared to when tissue is frozen prior to optical measurements^30^. Moreover, tissue fixation did not affect the lifetime of fluorescent proteins produced in HeLa cells^31^.

Direct monitoring of cell death at its early stage using intensity and lifetime autofluorescence is not new. Wang and colleagues monitored the NADH fluorescence lifetime in cultured Hela cells as well as in 143B osteosarcoma after inducing apoptosis^32^. NADH lifetimes were found to increase for the first 15 minutes of imaging and gradually decrease thereafter^32^. For intensity-based measurements, individual NADH and FAD fluorescence spectra did not seem to change greatly upon apoptosis or necrosis, but rather the redox ratio, $\frac{NADH}{NADH+FAD}$, tended to increase systematically after treatments. The difference in the response of NADH fluorescence intensity and NADH lifetimes to metabolic perturbations is probably due to the difference in NADH binding, where the former measurement is sensitive to Complex I binding, while time-resolved NADH fluorescence arise from multiple protein bindings^32^. The shape of the autofluorescence spectra is also known to shift or distort under conformational or environmental changes^32^. Therefore to identify any initial metabolic transformations that occur at the onset of disease, changes in the TPEF/SHG intensity (Fig. 4), spectral shape (Figs 1 and 2), and the fluorescence lifetimes (Fig. 5) were all used to investigate the influence of compressing the laser’s pulse width on tissue.

Endomicroscopic measurements on fluorescent standards were first performed to test the effect of pulse duration on the photophysical properties of fluorophores removed from the confounding effects of light attenuation in tissue. The characteristic fluorescence spectral shapes (Fig. 1(a,b)) and lifetime values (Fig. 1(c)) did not change with pulse duration, but had the desired effect of amplifying the two-photon fluorescence intensity. Moreover, the non-linear endoscopic measurements resulted in mean fluorescence lifetimes of 2.46 ± 0.019 ns and 3.89 ± 0.075 ns for Rhodamine B and fluorescein respectively, which are consistent with that reported in the literature (2.28–2.34 ns for Rhodamine B and 3.6–4.2 ns for fluorescein) under two-photon excitation^33,34^. Similarly, shortening the duration of the laser pulses did not alter the photophysical, metabolic and protein-binding states of endogenous fluorophores in *ex vivo* tissue (Figs 2 and 3 and Table 1). Despite tissue being a diffusive medium, the spectral shapes and the relative contribution of each fluorophore to the total fluorescence spectrum remained similar across all pulse durations (Fig. 2(c,d)). This is consistent with the study by Dunn *et al*., where the lateral resolution and the two-photon fluorescence intensity in tissue simulating phantoms were found to be largely independent on tissue optical properties (at both the excitation and emission wavelengths) up to a sampling depth of 250 µm^22^, and thus, the same conclusion is expected for excised fresh tissue. *In vivo*, tissue absorption and scattering will be accounted for by measuring the spectrally-resolved diffuse reflectance, which will be incorporated into the endomicroscope and is the subject of future studies. From this study, unmixing the fluorescence spectra and fitting the spectra to that of the five endogenous fluorophores indicate that there may be a peak unaccounted for in our fitting model around or below 400 nm (Fig. 2(c,d)). This may be attributed to autofluorescence or SHG from collagen crosslinks as the peak was even more prominent for meningioma and metastatic samples, but not evident in healthy tissue. In the meningioma and metastatic samples, modelling the additional SHG/autofluorescence signal as a Gaussian curve yielded better goodness of fit values and reduced the corresponding overall RMSE. Fitting the SHG/autofluorescence signal for GBM samples, on the other hand, resulted in poor fitting accuracy and was thus not included in the spectral fitting at 800 nm excitation. At 890 nm excitation, on the other hand, the SHG peak of collagen at 445 nm is well characterized in the total fluorescence signal, which was convolved in the TPEF images of Fig. 4 for GBM and metastatic tissue. The relatively high SHG signals due to increased collagen in meningioma and metastatic brain tissue are described extensively in the literature^34,35^. In both the endoscopic and imaging study, the two-photon intensities decrease according to ${\tau }_{p}^{-b}$, where ${\tau }_{p}$ is the pulse duration in femtoseconds and b = 1.21 ± 0.17, which is close to the theoretical value of 1^13^. Deviations from the theoretical trend has been attributed to the spectral broadening of the excitation beam as a result of shortening pulse width, where at ultrashort pulse durations, the spectral bandwidth can surpass the fluorophore’s spectral absorption bandwidth, significantly reducing the efficiency of two-photon absorption and in turn its emission^13,14^. This lower limit in pulse duration therefore depends on the fluorophore’s non-linear excitation spectrum^13,14^. In any case, the close to 1 exponent, b, suggests that the decays in the two-photon signals from all sampled tissue were not caused by photobleaching, but by the increase in the signal to noise ratio as predicted theoretically.

Pulse durations shorter than 100 fs at the focal plane were not always possible using the microscope setup due to the limited capabilities of the standard automated precompensation unit, but, the consistency in the fluorescence lifetime values for all four tissue types suggests that no photo-induced local heating, or changes in the pH or protein-binding states of the relevant biomolecules occurred as a result of compressing the pulses (Figs 3 and 5 and Table 1) for both point-based and microscopic imaging techniques. It is difficult to relate the fluorescence lifetime values recovered from the endoscope with those extracted from imaging for two reasons. First, FLIM data were collected at broader spectral bandwidths (see materials and methods section) where the lifetime values are derived from FAD (free) and from NADH (bound), while the point-based measurements were spectrally filtered to yield lifetime values of NADH, FAD, Lipopigments, and Porphyrin I. Second, the excitation wavelengths were different and thus the relative contribution of NADH and FAD to the overall FLIM signal at 890 nm will differ from that obtained with the endoscope at 800 nm. There is limited data on the fluorescence lifetimes of endogenous molecules in the neuronal tissue under two-photon excitation. The average fluorescence lifetimes in fixed meningioma tissue measured here agree with those found by Zanello and colleagues under two-photon excitation at 810 nm^35^. In the same study, the NADH lifetimes under one-photon excitation (at 405 nm) differed from that under two-photon excitation (810 nm)^35^. The only other study involving two-photon FLIM of human brain samples was that by Kantelhardt and colleagues who recovered average fluorescence lifetimes of 1.4 ns in parenchymal tissue and lifetimes of 2.1 ns in GBM^36^, under 750 nm, generally matching the values obtained here (Table 1). Incidentally, the relatively wide range of fluorescence lifetime values depicted on FLIM of healthy, metastatic, and meningioma tissue (Fig. 5) corroborated the need to perform a t-test on the endoscopic lifetime data for a single ROI rather than an ANOVA pooled from several ROIs, highlighting the benefit of wide-field quantitative imaging compared to point detection. Additionally, the small standard deviations relative to the mean values demonstrate that time-resolved fluorescence measurements are reproducible and can potentially be used as a quantitative biomarker (Table 1 and Fig. 5).

The photodamage induced by prolonged two-photon irradiation as a function of excitation power has been studied *in vitro*^14,19,37^, *ex vivo*^19,36,37^ and *in vivo*^16,18,37^, where tissue damage was assessed using different parameters. These mostly included evaluating the rate of photobleaching^14,19,37^, the rate of temporary photoenhancement followed by bleaching^16,19^, and the fraction of noticeable morphological damage observed on TPEF intensity images^19,37^. At first, the literature seems divided whether phototoxicity manifests itself as an overall decay in the fluorescence signal with a photobleaching rate having a 3^rd^ to 4^th^ order dependence on the mean laser power^38–40^, or whether phototoxicity is manifested as a sudden increase in the TPEF intensity followed by gradual photobleaching. Moreso, some studies report both phenomena within different regions of the same sample^18^. However, the confounding results can be explained by the different experimental conditions used, which can be stratified based on the irradiance at the site of measurement. Vogel *et al*., estimated a theoretical irradiance threshold for cell death at a repetition rate of 80 MHz to be 8 × 10^10^ *W/cm*^2^ ^41^. At such incident irradiance, plasma formation and cell blebbing from myelin sheath were simultaneously observed in fresh and frozen spinal tissue^41^, due to multi-photon absorption events. Photoenhanement was the main indicator of photodamage in Gali *et*. *al*’s *in vivo* and *ex vivo* mouse brain study^37^, where the threshold for photo-induced molecular damage ranged from 7 × 10^10^ to 13 × 10^10^ *W/cm*^2^ for poor and lipid-rich regions respectively^37^. The aforementioned irradiances are at least two orders of magnitude higher than what is used in our project. Nevertheless, the fact that the same manifestations of photodamage was evident for samples collected *in vivo*, in live cultured cells, as well as in frozen and fixed tissue at these high irradiances^37^, regardless of excitation pulse width, suggests that two-photon excitation induced damage is a photochemical effect rather than a physiological effect^19,37^, justifying as well, the use of fixed tissue in this study. Accordingly, if photo-induced damage had occured, fluorescence lifetime measurements would have indicated so.

The photobleaching study presented here involved irradiating tissue samples at two irradiances simulating the highest incident irradiance expected for future *in vivo* imaging with the endomicroscope as well as the highest irradiance currently used *ex vivo* with the multi-modal two-photon microscope at relatively short excitation pulse widths (100 fs). Since the TPEF signal decayed as a bi-exponential function (Fig. 6(a,b)), both oxygen mediated photobleaching and oxygen independent photobleaching pathways were implicated, which are the same pathways induced under one-photon excitation^42–45^. The faster photobleaching decay component has been attributed to the reaction of the fluorophore to molecular oxygen, while the oxygen independent pathway has been characterized with slower photobleaching rates^43^. Our samples were exposed to oxygen for at least 15 minutes prior to tissue fixation and another 15 minutes before acquiring two-photon images. Interestingly, the oxygen-mediated exponential decay caused 30% in the overall reduction of the TPEF signal under 2.2 × 10^8^ *W/cm*^2^, the maximum irradiation used on the multi-modal microscope, while it resulted in only 3% in the overall fluorescence decay under 1.0 × 10^9^ *W/cm*^2^, the maximum irradiation expected on the endomicroscope. This explains the faster photobleaching kinetics at 2.2 × 10^8^ *W/cm*^2^, where imaging of the same region of interest needs to be completed within the first few minutes. The rate of photobleaching does not only depend on the irradiance and the presence of oxygen, but on the local distribution of chromophores as well as the concentration of fluorophores^44^. The variation in the fluorophore concentration for different ROIs could further explain the difference in photobleaching kinetics at 2.2 × 10^8^ *W/cm*^2^ vs. 1.0 × 10^9^ *W/cm*^2^. Similar photobleaching kinetics was observed by Samkoe *et al*. where faster photobleaching occurred when live CV-1 kidney cells were irradiated with 3 × 10^7^ *W/cm*^2^compared to when the cultured cells were irradiated with 6 × 10^7^ *W/cm*^2^ ^43^. This was explained by the fact that oxygen consumption is slower under lower irradiance conditions and thus, the oxygen dependent regime lasts longer^43^. Additionally, in the same study by Samkoe *et al*., the oxygen-mediated photobleaching decay constant did not vary between the low and high irradiance conditions^43^, but rather the relative contribution of each photobleaching pathway depended on irradiance; this is also observed here (0.78 vs. 0.80 min^−1^).

Time-resolved fluorescence measurements did not change with prolonged irradiation (Fig. 6(c,d)), indicating no significant photochemical changes in the microenvironment during irradiation. The difference in the response of autofluorescence intensity and lifetime after prolonged two-photon excitation has also been observed in several photodamage studies^31,46,47^ and was attributed to the difference in each method’s sensitivity to the fluorophore’s biochemical state and microenvironment. In fact, Blinova *et al*., found that time-lapsed NADH fluorescence lifetime measurements under similar excitation and emission wavelengths used here, were sensitive to NADH binding to dehydrogenases, while fluorescence intensity based measurements were sensitive only to NADH associated with Complex I, providing another explanation for the observed differences^46^. A limitation of the photobleaching study is that TPEF and FLIM were detected across a broad spectral range (428–555 nm) and thus assessing individual endogenous fluorophores and their binding states was not possible. Therefore, under these conditions, one can conclude that the decay in the fluorescence intensity (Fig. 6(a,b)) is due to the reduction in the overall amount of endogenous NADH and FAD as a result of continued two-photon excitation, while the constant fluorescence lifetime values (Fig. 6(c,d)), corresponding only to bound NADH and free FAD, indicate that their local microenvironment, metabolic state, and their protein binding states did not change following prolonged two-photon excitation. Nevertheless, it could be possible that the origins of photobleaching and formation of oxidative species may not be detected with spectral range of the current detectors (~420–550 nm) and will be addressed in a pre-clinical brain tumour model prior to clinical intra-operative imaging.

It is important to reiterate that in the work presented here, compressing the excitation pulse duration did not result in photobleaching or any changes in the fluorophores’ properties when the mean laser power (1–2 mW), pixel dwell time (1.2 µs), and repetition rate (80 MHz) was maintained for 800 and 890 nm excitation wavelengths. Therefore, the precise effect of shortening excitation pulses will not only depend on pulse length but also on the very fluorophore, tissue volume, pixel dwell time, laser wavelength, the repetition rate and whether the excitation pulse is transform limited or chirped.

## Conclusion

The benefit of employing shorter laser pulses is well documented and further validated here on *ex vivo* brain tissue samples of different pathologies; The laser-induced tissue toxicity possible at these pulse durations due to thermal or photophysical effects have however, not been investigated despite the recent developments of compact ultrashort two-photon imaging modalities. This work studied the relationship between endogenous autofluorescence when tissue is two-photon excited at different pulse widths. Higher TPEF and SHG signals are achieved at shorter pulses without causing distortion in the spectral shape of individual fluorophores, nor any deviations from the expected inverse relationship between TPEF/SHG intensity and pulse duration. Similarly, the characteristic endogenous fluorescence lifetimes did not vary with decreasing pulse duration and with prolonged irradiation. Over time, photobleaching will eventually occur at a rate dependent on the excitation irradiance, but rather independent on the excitation pulse width. At the irradiances used here, the photobleaching curves demonstrate similar photobleaching pathways as described in conventional one-photon excitation and thus the same precautions in conventional pulsed laser-based imaging should be applied here at thresholds specifically defined for each two-photon imaging system and tissue type. Overall, these findings suggest that pulse durations compressed to 40–120 fs is sufficient to generate efficient TPEF and SHG signals without causing conformational, morphological or metabolic changes in neuronal tissue for a given mean laser power.

## Materials and Methods

The study protocol was approved by the human research institutional review board of the Sainte-Anne Hospital – University Paris Descartes (CPP Ile de France 3, S.C.3227). All methods were carried out in accordance with the relevant guidelines and regulations. Eight brain tissue biopsy samples were obtained from Sainte Anne Hospital’s neurosurgery department (Paris, France) with the informed consent of the patients and the hospital’s review board. The excised samples were fixed and classified as either Glioblastoma Multiforme, Meningioma grade I or II, Metastases, or healthy cortical tissue. The one healthy sample and three GBM samples were 150 µm thick, while the three meningioma and one metastatic tissue samples were sliced into 3 to 5 mm thick sections.

### Point spectral and lifetime fluorescence measurements with the endomicroscopic setup Instrumentation

**Figure 7:**
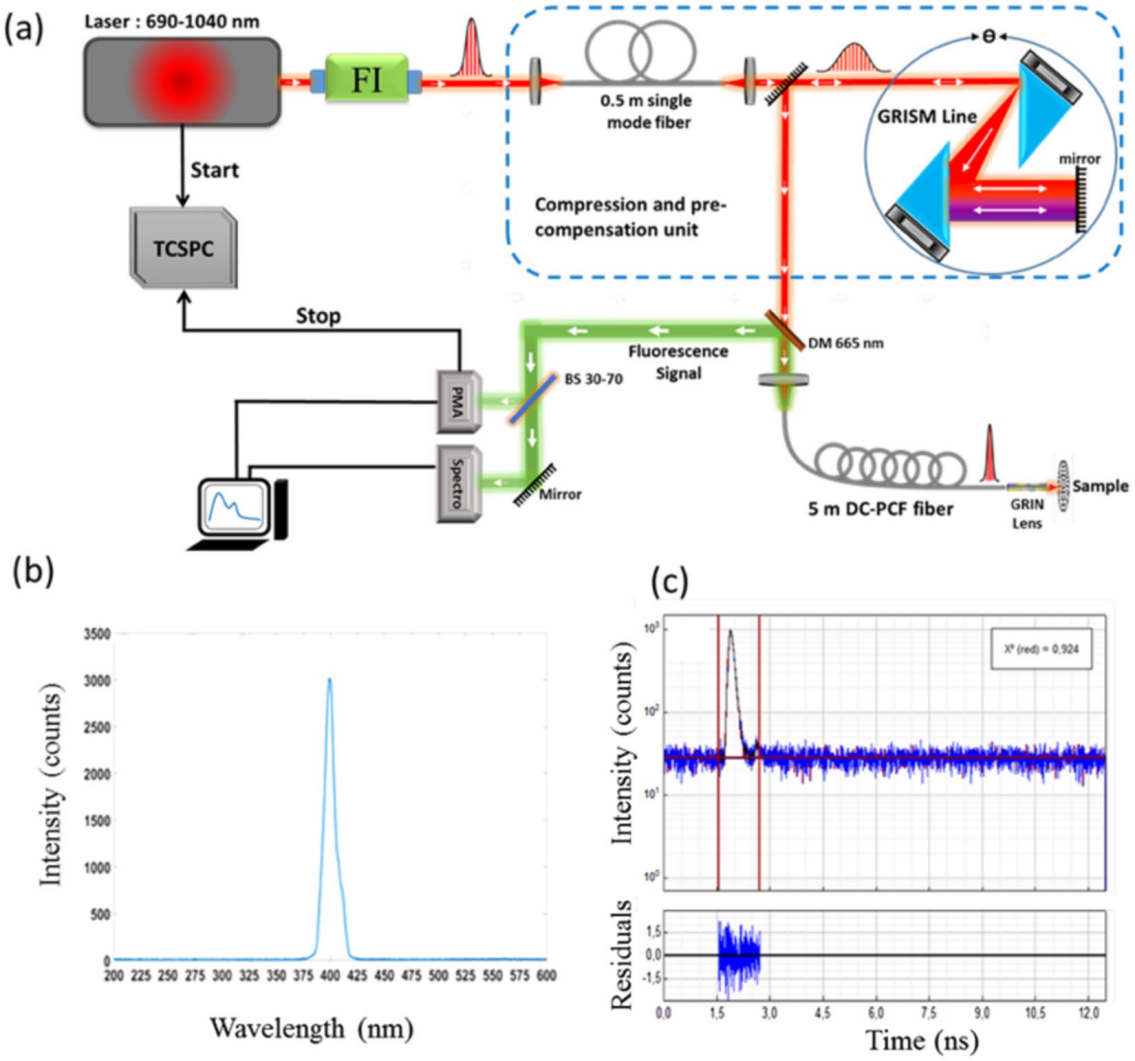
(**a**) Schematic of the endoscopic setup. TCSPC: time-correlated single photon counting, FI: Faraday Isolator, DM: dichroic mirror, DC-PCF: double-clad photonic crystal fibre, GRIN: gradient-index lens, BS: beam splitter, PMA: photomultiplier analyzer, and spectro: spectrometer; (**b**) raw SHG spectrum of urea; (**c**) fluorescence lifetime of urea fit to a Lorentzian function and its corresponding residual values. All components of the figure are produced by the authors.

The endomicroscopic setup built in-house is shown in Fig. 7(a). A Titanium: Sapphire laser source (Mai Tai DeepSee, Spectra-Physics, Santa Clara, CA, USA) is pumped with an average power of 2.4 W and is tunable from 690–1040 nm. The Faraday isolator (FI) prevents back reflection reaching the laser cavity. The 0.5 meter long single-mode fibre and the grating prism, GRISM, line together comprise the compression and precompensation unit, which corrects for second and third order dispersion effects, ensuring temporal confinement of the excitation beam and the generation of a Fourier-transform-limited pulse. The laser beam is then injected into the proximal end of a customized double-clad photonic crystal fiber (DC-PCF) characterized and described elsewhere^48^. Briefly, this DC-PCF has a central core diameter of 6.4 µm and an NA of 0.097 (at 800 nm), while its surrounding region is comprised of an air/silica microstructured region with a diameter of 40 µm. The outgoing beam is further focused by passing through a gradient-index (GRIN) lens, (GT-MO-080-018-AC-900-450, GRINTECH, Jena, Germany). The temporal pulse width can be modulated from 300 fs down to 25 fs at a repetition rate of 80 MHz by adjusting the distance between the two prisms of the GRISM line and the angle of the beam incident on the first prism. A dichroic mirror (DM) and a 660 nm low-pass filter separates the collected fluorescence signal from the incoming excitation beam. The beam splitter (BS) divides the fluorescence signal between spectrally (70%) and time-resolved detection arms (30%). A motorized filter wheel (450 ± 10 nm, 520 ± 10 nm, 550 ± 30 nm, 620 ± 10 nm and 680 ± 10 nm) and a hybrid photomultiplier detector assembly (PMA Hybrid 40 PicoQuant, Germany) collects the time-resolved fluorescence, while the spectrometer (QE Pro, Ocean Optics, France) collects the spectrally-resolved fluorescence signal with a spectral resolution of 1.5 nm and across the spectral range of 200 to 1000 nm. The characteristic SHG spectrum of urea in Fig. 7(b) (known to have negligible autofluorescence and a high SHG signal) obtained at the distal end of the endoscope signifies the fact that the DC-PCF does not generate background autofluorescence despite being 5 meters long. The instrument response function (IRF) of the system (Fig. 7(c)) from the same urea sample was found to be 13.5 ps after applying a Lorentzian fit. This signifies the system’s ability to resolve the fluorescence lifetimes of endogenous fluorophores from the IRF.

### Endomicroscopic measurements at different laser pulse durations

Two fluorescent dyes, Rhodamine B (Sigma-Aldrich) and Fluorescein (Sigma-Aldrich), both dissolved in water, were used as standards to validate their spectral shapes and lifetimes values with theory or data found in literature, prior to tissue measurements. For each standard, a drop of aqueous dye solution yielding a concentration of 10^−6^ M was placed on a microscope slide and positioned as shown in Fig. 7(a). For all measurements, the average laser power at the sample was 30 mW with a beam size of 0.1 mm and the excitation wavelength was 800 nm. Measurements were taken at pulse durations ranging from 40 fs to 150 fs. The lifetime decays were fitted to a mono-exponential decay function using the FluoFit software from PicoQuant and the characteristic lifetime value of each dye was extracted.

For the *ex vivo* fluorescence measurements, the fixed tissue samples were placed in the sample holder, and the same measurement variables as used for the fluorescent dyes were employed; the mean power of 30 mW with the range of pulse durations for the 800 nm excitation. For each pulse duration, and for each region of interest (ROI) of the sample, the time-resolved fluorescence lifetime decay profiles were collected and analyzed using FluoFit software to extract the lifetimes of four endogenous fluorophores: NADH, FAD, Lipopigments, and Porphyrin I. The fluorescence spectra, on the other hand, were first smoothed and then spectrally decomposed using a custom MatLab script to recover fluorescence spectra of these individual fluorophores including Porphyrin II. Per measurement, five ROIs were selected. For statistical analysis, a t-test was performed to test whether the lifetimes for each fluorophore were different in the five ROIs when measured at the same pulse duration. If the lifetimes were statistically similar (p > 0.05 and type II error, β, <0.1), a one-way ANOVA test was performed on lifetimes from all ROIs as a function of pulse duration. If the five ROIs resulted in significantly different lifetime values for the same pulse duration, a paired t-test was performed for each ROI as a function of pulse duration. In either case, significant differences between the mean lifetime values were determined for a p-value < 0.05.

### Multi-modal imaging with the multiphoton microscope Instrumentation

A multiphoton imaging platform for small animals (PIMPA) comprising the multi-modal multi-photon microscope (Leica TCS SP8 MP, Leica Microsystems, Wetzlar, Germany) and controlled through Leica’s acquisition software is the key instrument. The same Mai Tai Ti:Sapphire tunable laser source (690–1040 nm) equipped with an automated dispersion compensation unit was used. The maximum available power varied from 1.8 to 2.4 W for the different wavelengths. The laser pulse can be tuned between 70 fs and 300 fs at a fixed repetition rate of 80 MHz at the output of the cavity. A water-immersion objective was used (HCX IRAPO L 25x NA 0.95), and the field of view at a fixed focal depth was 433 µm by 433 µm for a working distance of 1.5 mm. Two external non-descanning hybrid detectors (Leica Hyd-RLD 2, Leica Microsystems, Wetzler, Germany) are added to the multiphoton microscope for enhanced two-photon detection, but spectrally-resolved two-photon fluorescence can be obtained as well. A 448 ± 20 nm band-pass filter (Semrock, FF01-448/20-25) is placed in front of the first detector for SHG detection, and a 520 ± 30 nm bandpass filter (Semrock FF01-520/35-25) is placed in front of the second detector for TPEF imaging. A time-correlated single-photon counting (PicoQuant TCSPC module, Berlin, Germany) is added to measure time-resolved fluorescence data. Lifetime images can be spectrally resolved by the two band-pass filters on the detection side.

### Two-photon imaging and FLIM at different laser pulse durations

For all *ex vivo* tissue measurements, the average power at the focal plane was set to 1.85 mW and 1.96 mW when the excitation wavelength was 800 nm and 890 nm respectively. The two wavelengths, 800 nm and 890 nm, were chosen for optimal TPEF and SHG detection respectively. The same wavelengths were used for FLIM as well. Prior to *ex vivo* measurements, the power at the objective for different pulse durations was measured. An acquisition of two-photon imaging of the samples required 500 ms, while FLIM required an acquisition time of 2–3 s per sample and the excitation pulse duration varied from 120 fs to 340 fs. TPEF and SHG images were analyzed on ImageJ, while fluorescence lifetime data collected at 448 ± 20 nm and 520 ± 30 nm were combined and fitted to a two-exponential decay function using the Symphotomie software provided by PicoQuant. For each tissue sample, five ROIs were chosen for fluorescence lifetime analysis at each pulse duration. Paired t-tests were performed for examining whether the recovered values differed significantly as a function of the excitation pulse duration. For the photobleaching study, TPEF and FLIM were acquired every minute for 20 minutes after 800 nm excitation at two irradiance conditions. In both cases, the pulse duration was set to 100 fs and the acquisition time was fixed at 20 seconds per image. In the first case, the average laser power was raised to 38 mW, and the TPEF images and FLIM were acquired after 800 nm excitation to emulate the maximum irradiance produced with the endomicroscope as suggested in our preliminary tests^25^; a mean laser power of 20–27 mW was more than sufficient to generate two-photon fluorescence in tissue at pulse durations of 40 fs^25^. For the same irradiance but for 100 fs pulse durations, this maximum laser power corresponded to 38 mW. The second case involved simulating the maximum average laser power used by the multi-modal microscope, which was 8 mW. The same set of measurements were performed on a different region of the sample but at this maximum laser power. Based on ongoing *ex vivo* measurements, laser powers higher than 8 mW resulted in saturating the sensitive hybrid detectors. In both cases, TPEF was first collected for twenty minutes, then an adjacent ROI was selected for FLIM. The same fitting routine used for the endoscopic lifetime measurements was performed at at each time point^48^.
